# A Review of Artificial Intelligence-Based Down Syndrome Detection Techniques

**DOI:** 10.3390/life15030390

**Published:** 2025-03-01

**Authors:** Mujeeb Ahmed Shaikh, Hazim Saleh Al-Rawashdeh, Abdul Rahaman Wahab Sait

**Affiliations:** 1 Department of Basic Medical Science, College of Medicine, AlMaarefa University, Diriyah 13713, Riyadh, Saudi Arabia; 2 King Salman Center for Disability Research, Riyadh 11614, Saudi Arabia; 3 Cyber Security Department, College of Engineering and Information Technology, Onaizah Colleges, Onaizah 56447, Al Qassim, Saudi Arabia; hazim@oc.edu.sa; 4 Department of Archives and Communication, Center of Documentation and Administrative Communication, King Faisal University, P.O. Box 400, Hofuf 31982, Al-Ahsa, Saudi Arabia

**Keywords:** genetic disorder, chromosomal abnormality, artificial intelligence, machine learning, facial images, ultrasound scans, genotyping array

## Abstract

Background: Down syndrome (DS) is one of the most prevalent chromosomal abnormalities affecting global healthcare. Recent advances in artificial intelligence (AI) and machine learning (ML) have enhanced DS diagnostic accuracy. However, there is a lack of thorough evaluations analyzing the overall impact and effectiveness of AI-based DS diagnostic approaches. Objectives: This review intends to identify methodologies and technologies used in AI-driven DS diagnostics. It evaluates the performance of AI models in terms of standard evaluation metrics, highlighting their strengths and limitations. Methodology: In order to ensure transparency and rigor, the authors followed the preferred reporting items for systematic reviews and meta-analyses (PRISMA) guidelines. They extracted 1175 articles from major academic databases. By leveraging inclusion and exclusion criteria, a final set of 25 articles was selected. Outcomes: The findings revealed significant advancements in AI-powered DS diagnostics across diverse data modalities. The modalities, including facial images, ultrasound scans, and genetic data, demonstrated strong potential for early DS diagnosis. Despite these advancements, this review outlined the limitations of AI approaches. Small and imbalanced datasets reduce the generalizability of the AI models. The authors present actionable strategies to enhance the clinical adoptions of these models.

## 1. Introduction

Down syndrome (DS) is a genetic condition showcasing the existence of an additional copy of chromosome 21 in an individual’s genetic markup [1]. It is one of the most prevalent chromosomal abnormalities, with the global incidence estimated to be around one in every 700 live births [1,2]. Individuals with DS exhibit specific physical characteristics, including unique facial features, short stature, and varied degrees of intellectual and developmental disabilities [3]. A comprehensive, precise, and early diagnosis is essential for providing prompt treatments and support for DS individuals and their families. In recent decades, medical and technological improvements have improved DS individuals’ life expectancy and quality of life [4]. A significant number of DS individuals are actively engaged in activities related to education, employment, and community involvement. Over the years, multiple prenatal and postnatal screening and diagnostic technologies have been developed, providing opportunities for parents and medical professionals to identify the existence of DS in the initial stages [5,6,7,8,9,10,11,12,13,14,15,16]. In the past, the diagnosis of DS was primarily accomplished by clinical observation of physical characteristics and certain developmental stages. Commonly, medical professionals examine for facial characteristics, hypotonia (low muscle tone), and developmental abnormalities [7,8,9,10]. However, the availability of genetic and biomarker-based diagnostics leads to a faster and conclusive diagnosis of DS. During prenatal screening, ultrasound imaging is an essential component in addition to genetic testing [12,13]. Additionally, in the first trimester, healthcare professionals evaluate nuchal translucency (the thickness of the fluid at the back of the fetal neck) to determine the likelihood of genetic disorders [14,15]. Table 1 summarizes DS phenotypes and health comorbidities. It emphasizes the importance of early diagnosis, addressing preventable and manageable health complications at different life stages.

Recently, artificial intelligence (AI) tools and techniques have supported radiologists and medical practitioners in producing more accurate diagnoses of subtle or complicated signs associated with DS [16]. In the field of biomedical research, the use of traditional ML methods, including logistic regression, decision trees, support vector machines (SVMs), and random forests (RFs) has become more prevalent. Data may be obtained in a variety of formats, including 2D ultrasound pictures, 3D/4D volumetric scans, and genetic sequences with biological signals [16]. Typically, ML methods depend on hand-crafted features [17,18,19]. Researchers or clinicians may independently detect biomarkers from an ultrasound image and process them using ML algorithms to predict DS. In the last decade, the healthcare industry has shifted toward deep learning (DL), a subset of ML using artificial neural networks with multiple layers to automatically learn characteristics from raw data [19,20]. For instance, convolutional neural networks (CNNs) have the potential to detect DS using ultrasound scans. Advanced architectures, including recurrent neural networks (RNNs) or transformers, are employed to analyze time-based signals like fetal heart rate monitoring. Researchers use DL algorithms to detect subtle patterns associated with DS, leading to earlier and more accurate diagnoses.

Agbolade et al. (2020) offered a review of DS diagnosis using facial features. They focused on theoretical advancements in DS diagnosis approaches [21]. The lack of detailed performance metrics across multiple datasets limits the generalizability of their conclusions. De Barros et al. (2023) presented a systematic review of automated DS tools and techniques [22]. This review’s findings are constrained by the homogeneity of datasets. The limited validation process minimizes the broader applicability of the presented techniques. Koul et al. (2023) investigated the use of AI techniques in diagnosing DS [23]. They presented the role of different diagnostic modalities, including medical imaging, genetic markers, and facial images, in DS diagnosis. However, the broader scope of this study limits an in-depth analysis of specific modalities. Vidhyasagar et al. (2024) evaluated DL strategies for early detection of DS through ultrasound imaging data [24]. They presented technical approaches without addressing the challenges of integrating these strategies into clinical workflows. Shafi et al. (2024) highlight the exponential growth of AI-based DS diagnosis techniques [25]. This study primarily focuses on the quantity of publications rather than the clinical impact of the research studies. These studies provide valuable insights into DS diagnosis using advanced technologies. However, a significant gap remains in understanding the generalization capabilities of existing models across diverse populations and clinical settings. In addition, these studies lack comprehensive evaluations of potential challenges, including algorithm biases and variability in clinical workflows.

The authors present a comprehensive evaluation of the AI model’s capability in diagnosing and managing DS. The study contributions are outlined as follows:

1. Exploring data types and technologies in diagnosing DS

The study investigates the diverse types of data sources for DS diagnosis. It maps the data modalities to specific diagnostic challenges. For instance, genetic data can present effective outcomes. However, they require high computational resources. In addition, the authors identify the underexplored data sources, including multimodal datasets.

2. Evaluating the performance of AI applications

The authors evaluate the effectiveness of AI applications in terms of accuracy, sensitivity, specificity, recall, precision, and F1 score across multiple studies. Using these metrics, they identify AI models, achieving higher diagnostic accuracy. In addition, they establish thresholds for reliable performance, guiding future research and clinical adoptions of AI tools.

3. Presenting the challenges faced by AI-driven diagnostic approaches

The authors investigate the challenges encountered during the development of AI applications. They present critical shortcomings, such as biases, interpretability challenges, and ethical concerns in applying AI in prenatal diagnostics.

4. Strategies for addressing biases and enhancing generalizability

The study emphasizes the significance of interpretability, visualizing the biomarkers influencing the AI model’s decision. It presents actionable solutions to promote equity and reliability in AI-driven diagnostics.

The remaining part of this study provides a structured analysis of AI-based DS diagnosis. Section 2 outlines the review methodology. It presents the systematic approach, identifying, evaluating, and synthesizing relevant studies. Section 3 reveals the datasets, AI models, and their respective contributions. The study implications, challenges, and future directions are presented in Section 4. Lastly, Section 5 concludes with a summary of the study’s key findings.

## 2. Review Methodology

The authors employ a structured methodology adhering to PRISMA guidelines to determine the role of AI in diagnosing and treating DS, focusing on techniques, datasets, outcomes, limitations, and challenges. They followed the PRISMA flow diagram to present the process of each stage of this review in order to ensure transparency and replicability. A systematic search was conducted across multiple academic databases. These databases were utilized to capture a broader range of research studies associated with DS diagnosis.

### 2.1. Research Questions

The authors framed the research questions in order to target critical aspects of AI-driven diagnostics, ensuring a comprehensive exploration of DS detection approaches. The research questions are as follows:

Research question 1 (RQ1): What types of data and technologies have been used to diagnose and treat DS?

This question explores diverse data, including facial images, genetic information, and ultrasound scans, to diagnose DS using AI techniques. It supports highlighting underexplored data sources, such as multimodal datasets integrating genetic and imaging data.

Research question 2 (RQ2): To what extent do AI applications perform in terms of evaluation metrics?

RQ2 investigates the performance of AI models, enabling a comparative analysis of AI methodologies. It determines a benchmarking framework for future research, identifying thresholds of acceptable accuracy for clinical adoption.

Research question 3 (RQ3): What challenges are faced by AI-driven diagnostic approaches?

This question explores the challenges in developing AI-based DS diagnostic approaches. It identifies the model’s potential to handle challenges in real-world generalizability. It outlines methodological trade-offs to support researchers in selecting appropriate techniques.

Research question 4 (RQ4): What strategies can be employed to reduce biases and improve the generalizability of AI models?

RQ4 focuses on strategies to improve the generalizability of AI models across diverse populations. It presents actionable solutions, promoting equity and reliability in AI-driven diagnostics. It identifies successful bias-reduction techniques for DS diagnosis.

### 2.2. Search Strategy

The authors cover diverse aspects, including medical imaging, symptom recognition, and clinical decision support systems. In addition, they delve into the ethical considerations, barriers to implementation, and the potential implications of AI-powered prenatal diagnosis. A systematic approach was developed to achieve a comprehensive review of relevant literature. The primary academic databases, including PubMed, IEEE Xplore, Scopus, and Web of Science, are utilized to extract cutting-edge AI approaches. A list of keywords and phrases was crafted to capture potential studies, minimizing unrelated outcomes. The keywords were selected based on their relevance to the RQs. The terms, including “down syndrome”, “artificial intelligence in healthcare”, “AI diagnosis tools”, “machine learning for genetic disorders”, “deep learning for down syndrome”, “AI-based pre-natal screening”, “clinical decision support systems for down syndrome”, “AI in medical imaging”, and “neural networks in diagnostic tools”, were used for extracting the studies. The authors employed Boolean operators (“AND” and “OR”) to refine the search. These operators are used to broaden the search, retrieving meaningful studies.

### 2.3. Inclusion and Exclusion Criteria

In order to effectively filter the articles, explicit inclusion and exclusion criteria were established. These criteria serve as a guideline for the selection process, ensuring the extraction of high-quality studies aligns with the RQs. Table 2 outlines the inclusion and exclusion criteria.

### 2.4. Screening and Selection Process

In order to identify the most suitable articles, the screening and selection procedure used a systematic, multi-stage approach. Initially, the authors conducted a title-screening procedure to exclude irrelevant articles. At this stage, studies focused on conventional diagnostic procedures and which did not make any reference to artificial intelligence were excluded. This process drastically reduced the article pool, allowing the articles to align with the review’s criteria for further evaluation. As an additional procedure, the authors examined the abstracts. This step aimed to verify that the studies adhered to the inclusion criteria. Finally, the authors conducted a full-text review of shortlisted studies to determine depth and quality. They considered rigorous research with comprehensive datasets and significant findings. This process assisted the authors in reducing the likelihood of including low-quality or irrelevant studies. With this multi-layered approach, the most pertinent studies were included for analysis.

### 2.5. Data Extraction and Critical Appraisal

The authors developed a standardized data extraction form to capture key information from each study. By leveraging this approach, they captured critical data, including DL algorithms, datasets, performance, strengths, and limitations. They considered feature extraction methods and the training and validation processes. A critical appraisal was conducted to evaluate the reliability of the studies, focusing on methodological rigor. Articles were examined for the availability of essential data, replicating the research using datasets and AI algorithms. The authors targeted potential biases, including limited dataset diversity and overfitting, leading to poor generalization. Lastly, ethical adherence was reviewed. This appraisal highlights the key areas requiring further research and improvement, guiding the development of reliable and inclusive AI-based DS detection approaches.

## 3. Results

**Figure 1:**
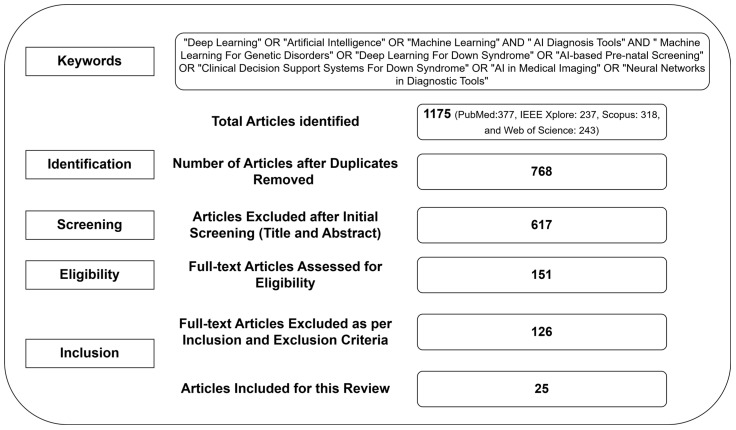
PRISMA flow diagram.

In this section, the authors reveal the findings of this review. The findings are categorized based on data modalities, showcasing the effectiveness of AI models in terms of evaluation metrics. Figure 1 summarizes the extraction process, providing a clear representation of the progression from article identification to final selection of studies. The initial search yielded a substantial total of 1175 articles. Based on the inclusion and exclusion criteria, the subsequent screening process excluded irrelevant articles. By meticulously filtering the literature, 25 studies were deemed suitable for inclusion into this review.

**Figure 2:**
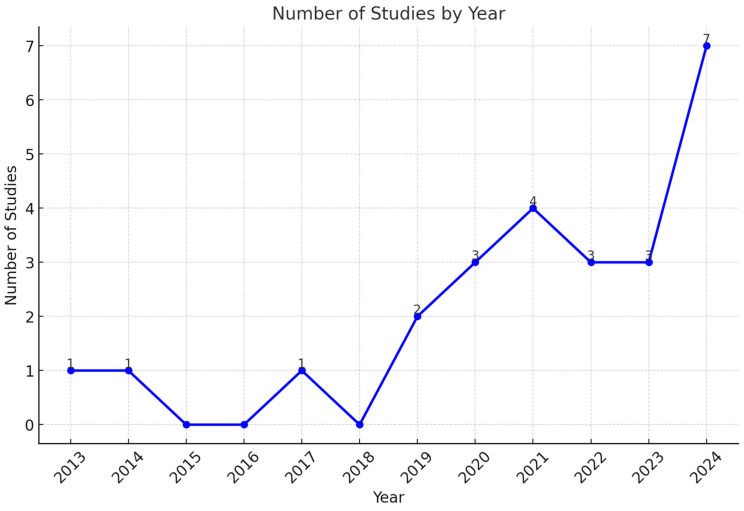
Timeline of research studies.

A chronology of research studies on the uses of AI in DS diagnosis is shown in Figure 2. This timeline highlights a considerable growth in academic interest over the course of the years. A single study was published per year between the years 2013 and 2017. Technical restrictions and the availability of datasets restricted the early attempts to integrate AI into DS diagnoses. Advancements in deep learning and machine learning technologies have been driven by continuous growth in research, representing a milestone in 2021. These developments are signs of increasing confidence in the ability of artificial intelligence to identify and resolve challenging diagnostic problems. The year 2021 reaches a high with four studies, indicating a surge of innovation. This may result from improved computing tools and a more extensive understanding of the usefulness of artificial intelligence in the medical field. However, the minor decline in 2022 and 2023, with three studies, may indicate difficulties in maintaining the rapid pace of research. These challenges may include restricted access to different datasets or limitations to clinical adoption.

In 2024, there was a significant increase in DS diagnostics approaches, achieving a total of seven studies. This increase is a result of developments in AI algorithms, collaborations across diverse fields, and the significant role of AI in medical diagnosis. It demonstrates the increased academic and multidisciplinary interest in developing effective AI-powered DS detection models. In addition, an increasing trajectory highlights the importance of maintaining research efforts in order to overcome current challenges in implementing AI applications in the healthcare sector.

### 3.1. Data and Technologies with Their Performance

**Figure 3:**
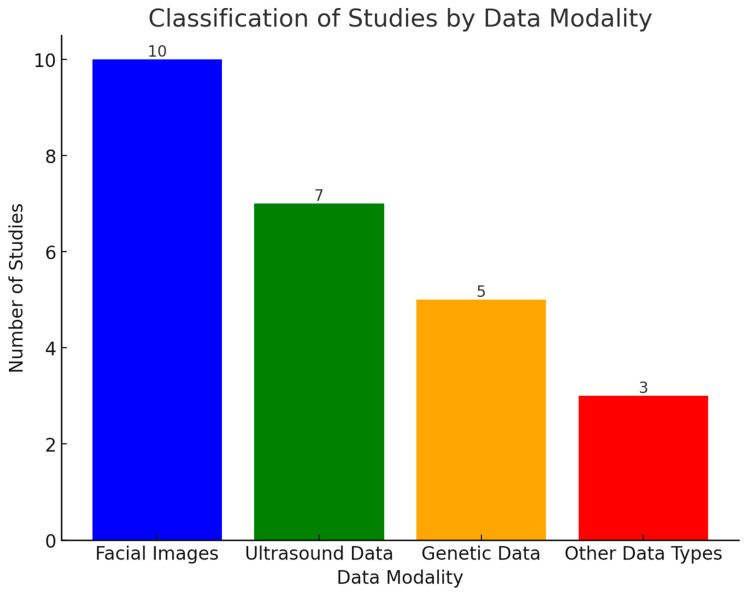
Distribution of research studies based on data modalities.

In this section, the authors address RQ1, RQ2, and RQ3. Data modalities, including facial images, ultrasound scans, and genetics data, are primarily utilized for DS diagnosis. The authors classify the studies based on the data sources. Figure 3 reveals the distribution of studies across different data modalities. It indicates the importance of facial images in postnatal diagnosis. The studies use AI-driven facial recognition to identify DS-associated phenotypic markers. Likewise, ultrasound scans emerge as a crucial modality in prenatal screening. The studies demonstrated the exceptional capability of ultrasound markers, enabling earlier identification of DS during pregnancy. A total of five studies highlights the significance of genetics data (molecular-level) diagnostics. By analyzing the genotyping array, AI models contributed to understanding the genetic basis of DS. Lastly, three studies of other data types reflect an underexplored area in DS diagnostics.

#### 3.1.1. Facial Images

Table 3 presents valuable insights into the advancements and challenges of the DS detection model using facial images. Each study produces a unique contribution towards DS detection, showcasing variations in methodologies and performance metrics. Zhao et al. (2013) [26] introduced a constrained local model to detect DS. They extracted geometric and texture features. They evaluated classifiers, including a support vector machine (SVM), k-nearest neighbor (K-NN), and random forest (RF). The synergy of geometric and texture features improved the model’s potential in determining DS patterns. However, the variation in image acquisition tools and illumination may introduce inconsistencies in the model’s outcomes. Zhao et al. (2014) [27] proposed an ensemble learning (EL)-based DS detection model. They applied feature selection and dimensionality reduction techniques to optimize the classifiers. The outputs of classifiers were an ensemble using majority voting, a pairwise fusion matrix, and statistical rules. The experimental findings highlight the reliability and flexibility of the EL approach. However, the EL approach may demand high computational requirements, limiting the model’s efficiency in real-time applications.

Mittal et al. (2020) [28] presented a robust DS framework, achieving reliable performance. They employed the AlexNet model for the feature extraction. An SVM model was used to classify the features into DS and normal classes. The model captured finer details through the extraction of unique facial regions, contributing to accurate classification. Pooch et al. (2020) [29] demonstrated the potential of integrating CNNs and an SVM classifier in detecting DS. They extracted features using the Inception-ResNet-V2 model. The features were classified using a linear SVM. A 10-fold cross-validation was employed to ensure robust performance. Qin et al. (2020) [30] introduced a deep CNN model-based DS detection approach. The approach involved a three-step pipeline: image preprocessing, facial recognition model, and fine-tuning DS syndrome identification.

Geremek and Szklanny (2021) [31] employed multi-task cascaded CNNs for face detection and alignment. They used seven DL models for the feature extraction. An SVM was used to classify the features. The model’s capability with mobile platforms enhances its potential for low-cost implementation. Porras et al. (2021) [32] presented a framework for genetic syndrome screening using facial images. Three neural networks models were used for image standardization, facial morphology, and estimation of genetic syndrome risks, respectively. Wang et al. (2022) [33] developed a DL framework using ResNet64 with a squeeze-and-excitation block for DS detection. They employed cross-loss training and transfer learning approaches to improve the model’s performance.

Islam and Shaikh (2024) [34] proposed a genetic disorder detection system that analyzes distinctive facial deformations associated with multiple genetic conditions. They employed a specialized attention mechanism, prioritizing critical features differentiating genetic syndromes. Raza et al. (2024) [35] proposed a novel framework integrating VGG16, non-negative matrix factorization, and light gradient boosting machine techniques. A K-fold cross-validation was used to evaluate the model’s performance.

#### 3.1.2. Ultrasound Scans

Table 4 provides an overview of AI models using ultrasound scans. It outlines key aspects of each study, highlighting the ability of AI models to extract and classify critical biomarkers. Yekdast (2019) [36] introduced a hybrid DS detection model integrating CNN and particle swarm optimization (PSO) models. The CNN model automates the feature extraction process, and PSO reduces the risk of model overfitting. Zhang et al. (2021) [37] introduced a DS screening tool. SVM and classification and regression tree (CART) algorithms were used for the feature classification. Z-score standardization was employed to ensure feature consistency. Thomas and Arjunan (2022) [38] developed a DL model for the early detection of DS. They automated the segmentation of the nuchal translucency (NT) regions. The AlexNet architecture was used to classify the extracted features. Zhang et al. (2022) [39] introduced Trisomy21Net to detect DS. A shallow CNN was employed with multiple dropout layers to control model overfitting. Class activation maps (CAMs) were used to improve the model’s intepretability. Tang et al. (2023) [40] employed the ResNet-18 architecture with advanced preprocessing and heatmap visualization techniques. They identified Trisomy 21, 18, and 13, using the ultrasound images. By leveraging the Grad-CAM technique, they provided interpretable insights, demonstrating the model’s versatility and robustness. Mavaluru et al. (2024) [41] proposed a DS detection model using enhanced feature extraction and segmentation techniques. Fuzzy gradient vector and snake edge techniques were used to identify the fetal weight irregularities. Reshi et al. (2024) [42] proposed a DL-based framework using fetal ultrasound images. The NT regions were extracted using a segmentation approach. An 11-layer CNN model was employed for feature classification.

#### 3.1.3. Genetics Data

Table 5 highlights the performance of AI models based on genetic data. Genetic data, including a genotyping array and other genomic datasets, play a significant role in detecting DS in the early stages. Feng et al. (2017) [43] presented a novel technique to enhance the performance of DS diagnosis using a genotyping array. They employed a CNN model to analyze chromosome single-nucleotide polymorphism (SNP) maps derived from Illumina genotyping arrays. The genetic information was transformed into visual data in order to enable the CNN to detect patterns and correlations within adjacent SNP sites. The model achieved an average accuracy of 99.3%. It surpassed SVM, random forests, and decision tree techniques. This study highlighted the potential of a non-invasive SNP genotyping approach. He et al. (2021) [44] introduced a DL-based DS detection model. They trained the model using the ensemble learning approach. In their study, 10-fold cross-validation was used for the internal validation. Goh et al. (2023) [45] developed a model to detect the genetic disorder. They utilized genomic deoxyribonucleic acid (DNA) samples to train and test the model. K-means clustering was used to categorize genomic wave patterns. Additionally, k-nearest neighbor (K-NN) was employed to classify the clustered features. Baldo et al. (2023) [46] presented an automated DS detection framework using genetic, diagnostic, clinical, and auxological data. They employed RF and gradient boosting machine (GBM) models for feature classification. Do et al. (2024) [47] utilized serum biochemical markers and ultrasound measurements for DS screening. They employed an ensemble learning approach for data classification and prediction.

#### 3.1.4. Other Data Types

Table 6 presents a detailed analysis of AI models utilizing diverse data types. The studies outline innovative methodologies using biochemical markers, brain imaging data, and video recordings. Li et al. (2019) [48] proposed a DS prediction model using multi-modality data. They extracted diverse indicators of DS. A cascaded ML framework was used to predict DS. In addition, 10-fold cross-validation was used for model evaluation. Mal’e et al. (2024) [49] employed generative models for extracting brain biomarkers associated with DS. They evaluated three cutting-edge models: variational autoencoders, reverse autoencoders, and diffusion models. Using these models, they identified biomarkers, including smaller cerebellum volumes, enlarged ventricles, and parietal lobe changes. A proprietary dataset, enriched by publicly available control datasets, was used to train and test the models. A novel histogram post-processing technique was introduced to improve the performance of variational and reverse autoencoders. The study focused on mid-sagittal plane MRI scans, potentially overlooking key information from 3D volumetric data. The diffusion models may face challenges in reconstructing anatomical features. Galindo-Lopez et al. (2024) [50] employed CNN–long short-term memory (LSTM) for analyzing video sequences. The dataset covers video recordings of 12 parent–child dyads during educational tasks. The convolutional layer for feature extraction was integrated with LSTM layers for temporal dependencies.

#### 3.1.5. Classified Findings of Reviewed Studies

Table 7 offers a comparative analysis of various DS diagnostic approaches, underscoring the potential of AI in refining DS diagnosis. Ultrasound image-based models achieve a sensitivity of 85–92%, resulting in an accuracy of 90–100%. However, these approaches face challenges in handling the complexities of images associated with operator dependency and variability in imaging equipment. Genetics-based approaches show sensitivity ranging from 61–96%, leading to high accuracy ranges of 66–99%. ML techniques, including RF, SVM, and deep neural networks, optimize genetics data analysis to identify crucial patterns, improving classification performance. AI-based facial image analysis achieves an overall accuracy of 88–99% with sensitivity (87–96%) and specificity (83–97%). This approach can be a non-invasive alternative for DS diagnosis. CNNs with attention mechanisms enhance feature extraction, achieving exceptional accuracy. Finally, other AI-driven approaches exhibit high accuracy (90–96%) and sensitivity of up to 95%, rendering them the promising DS diagnosing approach.

#### 3.1.6. Challenges Faced by AI-Powered DS Diagnostic Models

Based on RQ3, this review identifies the multifaceted challenges hindering the integration of AI in clinical workflows. The findings outline systematic and technical barriers, laying the foundation for developing effective and reliable AI diagnostics. Data imbalance and limited availability of data are the primary challenges in AI model development. In the context of DS, datasets with few positive instances tend to favor the majority class in models. The imbalance reduces sensitivity and increases false negatives, leading to undiagnosed patients. Privacy laws restrict data exchange and cooperation between organizations. Models validated in the controlled environments may not represent real-world performance, reducing their generalizability.

Models struggle to deal with diversity in clinical practices, imaging equipment, and patient demographics. The generalization of an AI model trained on high-resource healthcare data may be suboptimal in low-resource settings. AI biases typically reflect training dataset underrepresentation, resulting in inconsistent demographic outcomes. For instance, models may favor certain ethnicities, perpetuating healthcare disparities. The interpretability of AI models remains a primary barrier to their deployment in healthcare settings. Advanced models frequently predict without explaining decision-making processes. The absence of transparency lowers clinicians’ confidence and makes incorporating AI models into clinical workflows more difficult. Advanced AI models require substantial computational resources, high-performance hardware, and specialized expertise. These requirements restrict accessibility, especially in rural healthcare institutions and low-income countries.

### 3.2. Strategies for Reducing Biases and Improving Generalizability

Developing AI-enabled DS diagnostics demands an organized approach to guarantee robust, equitable, and effective outcomes. To maximize AI’s therapeutic potential, the challenges in data processing, model development, and workflow integration should be addressed. Successful DS diagnostics require diverse and high-quality datasets. The representativeness of artificial intelligence models may be improved by constructing extensive datasets comprising diverse populations across different demographics. In addition, approaches for data augmentation and synthetic data generation using generative adversarial networks (GANs) may be used to generate real-world samples in order to improve data diversity. The use of federated learning methodologies enables institutions to exchange model insights without disclosing sensitive patient data. Open data initiatives have the potential to facilitate the availability of large-scale datasets, promoting global collaboration.

**Figure 4:**
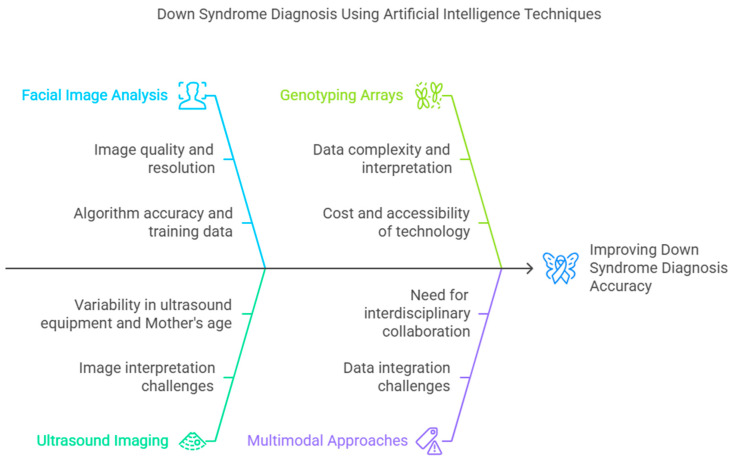
Strategies for improving DS diagnosis.

Improving the adaptability of AI models to diverse clinical contexts may be accomplished through the use of advanced approaches, including transfer learning and domain adaptation. The researchers can reduce the requirement for substantial retraining while retaining a high level of accuracy by fine-tuning pre-trained models. Furthermore, the use of fairness-aware algorithms guarantees equal performance across a wide range of populations. Over the course of model training, these algorithms make adjustments to account for potential biases, fostering fairness and inclusion. With the development of efficient and lightweight AI models, healthcare centers with limited resources may leverage the benefits of AI techniques. Figure 4 presents a comprehensive overview of challenges and strategies for improving AI-based DS diagnosis. To enhance the accuracy and reliability of the models, challenges associated with each modality should be addressed. Facial image analysis has gained significant traction in DS diagnosis. However, the image quality and resolution play a crucial role in achieving optimal results. High-resolution images can improve the identification of subtle facial dysmorphisms related to DS. The use of advanced DL models with robust data augmentation techniques can improve diagnostic accuracy. Genetics data or genotyping arrays offer a powerful DS diagnosis approach. However, this model poses challenges related to data complexity and interpretation. The cost and accessibility of genotyping technology can be a significant barrier in low-resource settings. Developing cost-effective genotyping technologies can facilitate broader adoption of this diagnostic approach. Ultrasound imaging is a widely used modality for DS screening. However, the variability in equipment and differences in maternal factors can influence the model’s performance. To overcome these challenges, the development of standardized protocols for image acquisition and preprocessing is essential. Multimodal approaches integrating facial images, genetics data, and ultrasound imaging can offer accurate and comprehensive DS diagnosis. Effective collaboration among healthcare professionals, geneticists, and radiologists, and AI specialists can lead to successful implementation of AI-based DS detection models.

The studies [51,52,53,54,55,56] emphasize the role of computer vision and AI in medical diagnostics analyzing multimodal data. Khalil et al. (2023) [51] demonstrated the capabilities of DL in detecting subtle image variations. By leveraging a fine-tuned CNN-based classified approach, early DS diagnosis using facial images can be improved. Abidin et al. (2024) [52] highlighted the importance of integrating multiple modalities in improving classification performance, reducing misdiagnosis, and enhancing generalizability. Cross-validation of facial features, ultrasound anomalies, and genotypic patterns can reduce false-positive and false-negative rates. Naqri et al. (2024) [53] and Iqbal et al. (2024) [54] introduced advanced DL approaches leveraging transformer-based noise reduction and adaptive ensemble learning techniques, addressing image-quality degradation and generalizability challenges. Enhancing image clarity of ultrasound scans and facial images can enable AI models to detect subtle morphological patterns. The integration of multiple AI models can reduce overfitting and improve sensitivity and specificity. A diversified model architecture can produce consistent performance across populations and medical imaging equipment. DL models are highly dependent on hyperparameter optimization and training strategies in order to guarantee their robustness and classification accuracy. Uppal et al. (2023) [55] and Abdulrazzaq et al. (2024) [56] explored different hyperparameter optimizers and self-supervised learning (SSL) to improve AI training with limited labeled data. Hyperparameter optimization enhances AI model performance by tuning model parameters, such as learning rate, batch size, and activation functions. A dropout rate of 0.3–0.5 balances learning while reducing overfitting in limited datasets. The activation functions, including the rectified linear unit, Swish, and Mish, are used to enable smoother gradient updates, leading to better facial and ultrasound feature extraction. Techniques including RMSProp and AdaDelta can handle non-stationary data distributions and adaptive learning rate adjustments. The incorporation of knowledge distillation, quantization, and edge AI can provide actionable insights into DS patterns. Data augmentation and GANs offer promising solutions to overcome the challenges in AI-based DS detection model development. Data augmentation techniques, such as rotation and flipping, scaling and cropping, color jittering, contrast adjustment, and Gaussian noise addition, can generate images with subtle variations, reducing overfitting to specific dataset distributions. Conditional GANs and deep convolutional GANs can be employed to generate synthetic DS samples. By simulating diverse facial structures and ultrasound variations, the model generalizability can be improved. Cycle GAN [57] and Pix2Pix [58] are widely used to enhance ultrasound image quality and noise reduction. These GANs can covert low-quality ultrasound scans into high-resolution images, improving fetal anomaly visualization.

Explainable artificial intelligence approaches are beneficial in improving the interpretability of AI models. Clinicians may gain an understanding of the logic behind AI predictions through techniques such as saliency maps, Shapley Additive exPlanations (SHAP), Local Interpretable Model-Agnostic Explanations (LIME), and Gradient-weighted Class Activation Mapping (Grad-CAM). By emphasizing key features associated with diagnostic findings, these tools can increase trust and confidence. In order to promote openness and enhance adoption among healthcare professionals, it is necessary to provide comprehensive documentation of model processes and the decision-making framework. SHAP assigns values to each feature, showing their influence in the model’s predictions. In DS diagnosis using genetic markers, SHAP can identify relevant biomarkers that contribute to classification. It can provide individualized explanations for ultrasound scan-based DS diagnosis. The visualization of DS risk scores can support clinicians in making informed recommendations for prenatal testing. LIME generates local surrogate models approximating the AI model’s behavior. It can support AI models in visualizing facial regions that contribute to classification. Visualizing the influential features, including the nasal bridge and epicardial folds, can assist clinicians in diagnosing DS using facial images. Similarly, Grad-CAM generates heatmaps showing crucial image regions associated with DS detection. In fetal ultrasound analysis, it can identify anatomical features that contributed to the diagnosis.

A systematic and multi-faceted approach is required to validate the AI-based DS detection model in diverse clinical settings. Multi-center data collection covering various hospitals, geographic regions, and diverse demographic groups is essential to train and test the model. To evaluate the model’s performance in real-world settings, external validation is crucial. Cross-population performance analysis is another key aspect to identify disparities in the model’s performance. Regulatory compliance frameworks include the Health Insurance Portability and Accountability Act (HIPAA) and the General Data Protection Regulation (GDPR).

Model accuracy and relevance may be maintained by automated data and technological updates. Version control systems manage updates and ensure clinicians use the recent models. In addition, hybrid methods, combining classic rule-based systems with modern AI techniques, provide a balance between simplicity and performance. Future studies should emphasize the execution of trials in real-world settings. By validating AI models in a variety of clinical settings, their reliability and applicability can be guaranteed. Additionally, these experiments may highlight operational challenges, including integration with electronic health record systems or healthcare provider interfaces. To overcome the challenges in implementing effective AI-based DS diagnosis, interdisciplinary collaboration is essential. By integrating the viewpoints of AI, genetics, healthcare, and bioinformatics professionals, advanced technology and therapeutically appropriate solutions can be presented. Diversity in viewpoints assists in recognizing gaps and generating comprehensive DS diagnostic models. For instance, physicians may help build AI models compatible with clinical processes, while ethicists can handle privacy, consent, and algorithmic fairness.

## 4. Discussions

The study findings provide substantial improvements in understanding the uses of advanced AI tools and techniques in the diagnosis and management of DS. The study covers the gaps in the existing body of knowledge and offers an extensive foundation for the advancement of DS diagnosis. Additionally, it offers practical implications, enhancing diagnostic accuracy and addressing existing limitations. The authors present an extensive representation of the data types and technologies utilized in DS diagnosis. The diagnosis procedure relies heavily on multiple data modalities, including facial images, genetic information, and ultrasound scans. Each modality has its own set of advantages and disadvantages. For instance, facial images were utilized in postnatal diagnosis, extracting DS phenotypes. In contrast, ultrasound imaging is essential to prenatal screening, detecting nuchal translucency. Extensive computing resources and expertise are essential in order to efficiently utilize genetic databases, including SNP genotyping arrays.

The authors address RQ1 through the investigation of DS diagnostics technologies. Based on the categorization of various modalities and their alignment with diagnostic problems, the study highlights the multifaceted nature of AI applications for DS diagnosis. The studies vary significantly in dataset compositions, reflecting the data diversity in AI-based DS diagnosis. Dataset sizes ranging from 100 to over 86,000 samples were used to train and test the models. The facial images-based models relied on high-resolution images for optimal classification performance. Ultrasound-based models required 300–4953 fetal scans to reinforce their high diagnostic potential. Genetics-based diagnostic models utilize datasets ranging from 378 to 86,142 samples. Facial image analysis employed CNNs, achieving accuracy rates between 88–99% with an AUROC value exceeding 0.95. Similarly, ultrasound imaging-based models achieved accuracy values ranging from 90–100%, with specificity reaching 100%, demonstrating AI’s potential in enhancing early-stage fetal assessment. These models minimize human error in nuchal translucency measurements. Genetics data have been instrumental in analyzing gene expressions and detecting DS with high accuracy. This review draws attention to the integration of multiple sources of data in order to improve diagnostic accuracy and dependability.

In line with RQ2, the authors investigated the efficacy of AI applications based on evaluation criteria. They offered actionable insights into the reliability of AI technologies using a comprehensive analysis. They identified levels of accuracy acceptable for clinical application and knowledge gaps in existing DS diagnosis literature. For instance, the review highlights the demand for larger datasets containing diverse data in order to improve precision and reduce biases. Similarly, DL models using images produced exceptional outcomes in terms of diagnostic accuracy, while ML classifiers have shown promise in the field of genetic data analysis. However, the authors noticed certain limitations, including limited sensitivity in small and imbalanced datasets, leading to increased rates of false-negative diagnoses. These findings are essential to guaranteeing accurate implementation of AI-driven diagnostic systems.

In response to RQ3, the authors analyzed the challenges in developing AI-powered diagnostic methods. This study highlights notable limitations, including the presence of biases due to training and testing of AI models on similar datasets, challenges in interpreting the outcomes, and ethical concerns associated with application of AI in prenatal diagnosis. These findings are essential in order to guide academics and medical professionals toward the implementation of AI models to produce effective and equitable solutions. The authors addressed RQ4 by presenting effective strategies for bias reduction and model generalizability. This emphasizes the need for real-world validation of artificial intelligence models across diverse clinical and demographic scenarios, ensuring their efficacy and applicability in various circumstances. GANs provide innovative solutions to data scarcity by generating synthetic images. These strategies are highly significant in handling critical barriers in real-world implementations. Table 8 highlights the research gaps and future research directions for DS diagnosis.

This study discussed cutting-edge DS diagnostic approaches. However, it has a few limitations, influencing the scope and applicability of its findings. The reliance on published studies may exclude valuable insights from gray literature, including industry reports and ongoing experimental works. This literature may cover practical implementations, leading to understanding the significance of AI in diagnosing DS. Excluding non-English studies may restrict the diversity of this study’s findings. Linguistic bias may overlook culturally specific insights, contributing to developing globally applicable DS diagnostic models. As AI technologies rapidly evolve, some conclusions may become outdated. In order to overcome these limitations, regular updates are essential to identify the latest advancements and reassess the findings. This approach can determine the emerging trends in DS diagnosis.

## 5. Conclusions

This review underscores the significance of AI techniques in DS diagnosis. It offers crucial insights into data modalities, methodologies, and clinical implications of AI models. Using facial images, ultrasound scans, and genetic data, AI models deliver promising outcomes. These advancements represent a paradigm shift in DS diagnostics. However, this review highlights the significant limitations of AI techniques, preventing widespread clinical adoption. This review serves as a foundation for academics, clinicians, and policymakers by providing a comprehensive analysis of existing research. AI-driven models can potentially transform prenatal and postnatal treatment, improving the DS individual’s quality of life. Addressing dataset imbalance, limited demographic diversity, and lack of real-world validation is essential to enhance the performance of AI-powered DS diagnosis. In order to establish trust and ensure equitable healthcare delivery, it is necessary to address ethical considerations associated with data privacy. The development of an extensive dataset, multimodal methods incorporating facial images, ultrasound scans, genetics data, and validations in diverse clinical contexts should be the highest priorities for future research. Despite its comprehensive scope, this review has certain limitations. The dependence on published studies may result in publication bias, overlooking valuable insights from gray literature and unpublished data. In addition, this review emphasized English-language articles, excluding studies published in other languages. By addressing these limitations, future studies can provide a holistic understanding of AI-driven DS diagnostics.

## Figures and Tables

**Table 1:** DS phenotypes and age-specific health comorbidities.

Age Group	Phenotype Features	Common Health Comorbidities
Childhood (0–12 years)	Intellectual disability, delayed motor development, and hypotonia	Congenital heart disease, hearing loss, and gastrointestinal disorders
Young adulthood (13–35 years)	Short stature, mild to moderate intellectual impairment, and speech difficulties	Obesity, obstructive sleep apnea, and early-onset Alzheimer’s
Late adulthood (36+ years)	Premature aging, cognitive decline, and osteoporosis	High risk of Alzheimer’s disease and increased cardiac issues

**Table 2:** Inclusion and exclusion criteria.

Inclusion Criteria	Exclusion Criteria
Peer-reviewed studies evaluating AI-based DS diagnostic approaches	Studies focused solely on non-AI diagnostic approaches
Studies encompass medical imaging and genetic data analysis using ML tools and techniques	Articles lacking quantitative metrics
Research addressing AI implementation into clinical workflows	Non-peer-reviewed studies
Studies presenting quantitative outcomes	Non-English studies and research with incomplete data
Article published in English	

**Table 3:** Characteristics of AI models based on facial images.

Authors	Dataset	Method	Performance	Strengths	Limitations
Zhao et al. (2013) [26]	100 frontal facial images; ethnicity: not specified; and location: not specified	SVM, Linear SVM, K-NN, and RF	Accuracy = 94.6%, sensitivity = 95.5%, and specificity = 92.5%	Non-invasive methodology and innovative feature extraction	Limited dataset, non-standardized conditions, and limited validation
Zhao et al. (2014) [27]	130 frontal facial images; ethnicity: Caucasian, African, and Asian; and location: National Medical Center, Abu Dhabi, United Arab Emirates	SVM, Linear SVM, RF, and linear discriminant analysis	Accuracy = 96.7%, sensitivity = 93.3%, specificity = 92.8%, and area under the receiver operating characteristic curve (AUROC) = 0.996	Comprehensive feature set, diverse dataset, and validation across classifiers	Computational complexities, limited focus on real-world testing, and image variability
Mittal et al. (2020) [28]	1706 images; ethnicity: multinational; and location: not specified	AlexNet with SVM	Accuracy = 98.5%, sensitivity = 96.2%, and specificity= 95.7%	Advanced feature extraction, scenario analysis, and focus on regional features	Gender-based variance, dependency on pre-trained models, and limited dataset
Pooch et al. (2020) [29]	170 images; ethnicity: Brazilian; and location: Rio Grande, Brazil	Inception-ResNet-V2 and Linear SVM	Accuracy = 94.0%, sensitivity = 95.0%, and specificity = 92.0%	Scalability potential and framework comparisons	Lack of a public dataset, non-standardized image acquisition, and relatively small dataset
Qin et al. (2020) [30]	405 images; ethnicity: Chinese; and location: China	Deep CNNs, SVM, and K-NN	Accuracy = 95.87%, sensitivity = 93.18, and specificity = 97.40%	Advanced deep CNN, effective use of transfer learning, and robust preprocessing	Dataset bias, limited clinical validation, and lack of model’s interpretability
Geremek and Szklanny (2021) [31]	2101 images; ethnicity: European; and location: Poland	Arcface, Deepface, FaceNet, other pre-trained CNNs, and SVM	Accuracy = 96%, sensitivity = 94.9%, and specificity = 92.1%	Generative capability and compatibility with mobile platforms	The limited scope of geometric analysis and demographic bias
Porras et al. (2021) [32]	1400 images; ethnicity: multinational; and location: not specified	Three neural networks models for DS risk identification	Accuracy = 88%, sensitivity = 90%, and specificity = 86%	Robust model, supports generalizability, and use of three distinct models	Limited validation and dependence on phenotypic features
Wang et al. (2022) [33]	490,000 images of 10,000 different individuals; ethnicity: Chinese; and location: China	ResNet 64 with squeeze-and-excitation block	Accuracy = 93.5%, sensitivity = 87.0%, and specificity = 83.2%	Cross-loss training and robust preprocessing approach	High resource requirements and demographic biases
Islam and Shaikh (2024) [34]	408 images; ethnicity: multinational; and location: multiple locations	CNNs with an improved attention mechanism	Accuracy = 95.4%, sensitivity = 88.1%, and specificity = 86.2%	Integrated attention mechanism and robust data preprocessing	Limited generalizability and high computational demands
Raza et al. (2024) [35]	3009 images; ethnicity: Middle Eastern; and location: Saudi Arabia	VGG16 and light gradient boosting machine	Accuracy = 99.0%, sensitivity = 92.3%, and specificity = 91.5%	Highly effective ensemble approach and effective feature extraction	Dependence on pre-trained model may reduce the model’s performance

**Table 4:** Characteristics of AI models based on ultrasound scans.

Authors	Dataset	Method	Performance	Strengths	Limitations
Yekdast (2019) [36]	300 images; mother’s age range: 28–35 years; ethnicity: Iranian; and location: Iran	CNN, PSO, and multi-layer perceptron-based classification	Accuracy = 99.38%, sensitivity = 92.1%, and specificity = 94.3%	PSO-based hyperparameter tuning and adaptation to diverse medical imaging data	Single dataset source and requirement of substantial computational resources
Zhang et al. (2021) [37]	4953 images; mother’s age range: 19–35 years; ethnicity: Chinese; and location: Jilin University, China	SVM and AdaBoost classification	Accuracy = 100%, sensitivity = 91.5%, and specificity = 94.3%	Integration of CART and AdaBoost and exploration of T21 risk cutoff thresholds	Reliance on serum markers and large computational resources requirements
Thomas and Arjunan (2022) [38]	100 images; mother’s age range: 19–31 years; ethnicity: Indian; and location: India	VGG-16 and AlexNet-based feature classification	Accuracy = 91.7%, sensitivity = 85.7%, and specificity = 100%	Speckle noise reduction approach and fine-tuned AlexNet-based feature classification	Small dataset and requirement of extensive preprocessing
Zhang et al. (2022) [39]	822 images; mother’s age range: 19–35 years; ethnicity: Chinese; and location: China	A shallow CNN with dropout layers	Accuracy = 90.5, sensitivity = 92.5%, and specificity = 90.6%	Robust data augmentation and visualization techniques	Reliance of image quality and limited dataset size
Tang et al. (2023) [40]	1120 images; mother’s age range: 19–35 years; ethnicity: multinational; and location: multiple locations	ResNet-18 with Grad-CAM heatmap technology	Accuracy = 93.0%, sensitivity = 89.0%, and specificity = 96.0%	Identification of key biomarkers using Grad-CAM heatmap visualization, a technique in TorchCAM 0.3.	Lack of external validation and quality of images may influence the model’s performance
Mavaluru et al. (2024) [41]	1075 images; mother’s age range: 28–35 years; ethnicity: Indian; and location: India	Feature extraction and segmentation approaches with SVM	Accuracy = 98.2%, sensitivity = 90.7%, and specificity = 92.8%	Integration of fuzzy gradient vector flow and wavelet-based segmentation	Limited generalization and segmentation challenges
Reshi et al. (2024) [42]	1120 images; mother’s age range: 28–35 years; ethnicity: Middle Eastern; and location: Saudi Arabia	CNN with dropout layers	Accuracy = 94.5%, sensitivity = 91.8%, and specificity = 94.2%	Focus on early pregnancy and comprehensive framework	High computational requirements

**Table 5:** Characteristics of AI models based on genetics data.

Authors	Dataset	Method	Performance	Strengths	Limitations
Feng et al. (2017) [43]	378 samples (only 63 DS samples); ethnicity: Chinese; and location: China	Customized CNN model based on genotyping array	Accuracy = 99.3%, sensitivity = 91.5%, and specificity = 88.6%	Dual-branch CNN design for visualizing feature maps	Relatively small sample size, limiting the model’s generalizability
He et al. (2021) [44]	86,142 individuals’ data; ethnicity: Chinese; and location: China	RF and SVM model	Internal validation: accuracy = 66.7%, sensitivity = 59.8%, and specificity = 61.3%; external validation: accuracy = 85.2%, sensitivity = 72.6%, and specificity = 73.8%	High predictive performance and robust generalization	Population specificity and data imbalance
Goh et al. (2023) [45]	742,750 SNP markers and 138 copy number variation (CNV)-related chromosomal disorders; ethnicity: Korean; and location: Seoul, South Korea	K-means clustering and K-neural networks	Accuracy = 100%, sensitivity = 95.8%, and specificity = 97.6%	Robust validation and enhanced CNN detection	Limited detection scope and dependence on genomic wave mitigation
Baldo et al. (2023) [46]	106 individuals; ethnicity: Italian; and location: Italy	RF and gradient boosting model (GBM)-based classification	Accuracy = 67%, sensitivity = 61.4%, and specificity = 60.7%	Innovative preprocessing, feature importance analysis, and robust classification model	Complexity of data inter-relationships and bias in age-related variables
Do et al. (2024) [47]	1035 individuals; ethnicity: Vietnamese; and location: Vietnam	RF and GBM-based classification	Accuracy = 90%, sensitivity = 83.2%, and specificity = 81.5%	Integration of multi-modality data and adaptations to diverse populations	High computational resources and dependence on data quality

**Table 6:** Characteristics of AI models based on other data types.

Authors	Dataset	Method	Performance	Strengths	Limitations
Li et al. (2019) [48]	100,252 features; ethnicity: Chinese; and location: China	Isolation forest model for detecting anomalies and refining predictions	Accuracy = 95.1%, specificity = 93.2%, and sensitivity = 95.0%	Efficient data imbalance handling and comprehensive feature selection approaches	Heavily relies on biochemical and ultrasound markers
Mal’e et al. (2024) [49]	Brain MRI scans, a total of 2113 images from multiple repositories, were used to train the model; ethnicity: Spanish; and location: Spain	Variational autoencoders and diffusion models were used to determine the subtle variations in MRI scans	Accuracy = 96.1%, specificity = 97.1%, and sensitivity = 93.0%	Biomarker discovery and quantitative volumetric analyses of subcortical structures	Relying on mid-sagittal 2D MRI scans may overlook critical spatial information
Galindo-Lopez et al. (2024) [50]	12 parent–child interaction video recordings; ethnicity: Mexican; and location: Mexico	CNN–LSTM model	Physical approach: accuracy = 92.1%, specificity = 90.1%, and sensitivity = 91.1%; verbal expression: accuracy = 90.96%, specificity = 88.1%, and sensitivity = 86.2%	Innovative hybrid architecture and high accuracy in capturing physical activities	Imbalanced datasets, camera perspective dependence, and verbal expression challenges

**Table 7:** General characteristics of DS diagnostic approaches.

Data Type	Sensitivity	Specificity	Accuracy	Risk Level
First-trimester ultrasound images	85–92%	94–100%	90–100%	Low
Second-trimester ultrasound images				Low
Genetics data	61–96%	60–97%	66–99%	Moderate
Facial images	87–96%	83–97%	88–99%	Low
Other	86–95%	86–97%	90–96%	Moderate

**Table 8:** Research gaps and future research directions.

Research Gaps	Future Research Directions
Limited demographic diversity, leading to biases in model predictions	The multinational dataset contains increased dataset representation across different ethnicities
Reduced model’s sensitivity and specificity in real-world clinical applications	Developing domain adaptation techniques and conducting multi-center external validations
Focused on single modality [facial images, ultrasound scans, or genetics data]	Building multimodality-based AI models, improving diagnostic accuracy and robustness
Requirement of significant computational resources, limiting deployment in resource-constrained healthcare settings	Exploring lightweight architectures for implementing cloud-based AI solutions
Lack of decision-making explanations	Integration of explainable AI techniques to enhance transparency
Different studies used varied evaluation metrics	Establishing standardized benchmarks and reporting guidelines
