# Correction: Shaikh et al. A Review of Artificial Intelligence-Based Down Syndrome Detection Techniques. *Life* 2025, *15*, 390

**DOI:** 10.3390/life15071152

**Published:** 2025-07-21

**Authors:** Mujeeb Ahmed Shaikh, Hazim Saleh Al-Rawashdeh, Abdul Rahaman Wahab Sait

**Affiliations:** 1 Department of Basic Medical Science, College of Medicine, AlMaarefa University, Diriyah 13713, Riyadh, Saudi Arabia; 2 King Salman Center for Disability Research, Riyadh 11614, Saudi Arabia; 3 Cyber Security Department, College of Engineering and Information Technology, Onaizah Colleges, Onaizah 56447, Al Qassim, Saudi Arabia; hazim@oc.edu.sa; 4 Department of Archives and Communication, Center of Documentation and Administrative Communication, King Faisal University, P.O. Box 400, Hofuf 31982, Al-Ahsa, Saudi Arabia

**Additional Affiliation**

In the published publication [1], there was an error regarding the affiliation for Mujeeb Ahmed Shaikh. In addition to affiliation 1, the updated affiliations should include: “King Salman Center for Disability Research, Riyadh 11614, Saudi Arabia”. The authors state that the scientific conclusions are unaffected. This correction was approved by the Academic Editor. The original publication has also been updated.
